# Treatment of Charcot Neuroarthropathy and osteomyelitis of the same foot: a retrospective cohort study

**DOI:** 10.1186/s12891-017-1818-4

**Published:** 2017-11-16

**Authors:** Martin Berli, Lazaros Vlachopoulos, Sabra Leupi, Thomas Böni, Charlotte Baltin

**Affiliations:** Department of Orthopedics, Balgrist University Hospital, University of Zurich, Forchstrasse 340, -8008 Zurich, CH Switzerland

**Keywords:** Charcot, Osteomyelitis, Amputation, Antibiotic, Treatment

## Abstract

**Background:**

We evaluated treatment of osteomyelitis in the foot in the presence of Charcot neuroarthropathy, a devastating condition with progressive degeneration and joint destruction. We hypothesized that there was a difference in (1) amputation rate, (2) amputation level, (3) duration of antibiotic therapy, and (4) duration of immobilization for treatment of osteomyelitis within versus outside the Charcot zone.

**Methods:**

Forty patients (43 ft) diagnosed with Charcot neuroarthropathy and osteomyelitis of the same foot were retrospectively analyzed. Some patients were successfully treated for osteomyelitis at different sites on the same foot at different times, thus 60 cases of osteomyelitis were identified in 40 treated patients. Cases were divided according to osteomyelitis localization: Group 1 had osteomyelitis outside the active Charcot region; Group 2 had osteomyelitis within the active Charcot region.

**Results:**

Male patients (*n* = 29; mean age 58.2, range 40.1 to 77.5 years) were younger than female patients (*n* = 11; mean age 70.4, range 51.4 to 87.5, *p* = 0.02 years). Amputation rate was 52% overall (26/40 patients; 26/43 ft): 63% of 30 Group 1 cases and 40% of 30 Group 2 cases (*p* = 0.09). Amputation level (*p* = 0.009), duration of antibiotic treatment (*p* = 0.045) and duration of immobilization (*p* = 0.01) differed significantly between the groups.

**Conclusions:**

Osteomyelitis within the Charcot region is associated with a higher level of amputation and longer durations of antibiotic therapy and immobilization. Osteomyelitis outside and within the Charcot affected region should be considered separately. If osteomyelitis occurs outside the active Charcot region, primary amputation may be preferred to internal resection.

**Level of Evidence:**

Retrospective cohort chart review study.

## Background

Charcot neuroarthropathy (CN), or diabetic neuropathic osteoarthropathy with progressive degeneration and joint destruction as a consequence of any condition resulting in decreased peripheral sensation [1], is a rare but devastating complication. The most common cause of CN is diabetes mellitus; other causes include alcoholism, vitamin B12 or folic acid deficiency, intravenous drug use, late stage syphilis, syringomyelia, and multiple sclerosis. While the precise pathogenesis of CN remains controversial, it is undoubtedly multifactorial [2, 3]. The current theory of Charcot pathogenesis combines both neurotraumatic and neurovascular aspects [3–6].

Elements of osteopenia, bone hyperemia, instability, muscle weakness, and loss of protective sensation place the limb at risk for developing neuropathic bone and joint changes [7]. When the compromised foot experiences trauma and the injury remains unrecognized, the cascade of events that subsequently ensues will often result in neuropathic fractures, subluxation or osteoarthropathy [8], and severe foot deformity. If an ulcer is also present, and the bone can be palpated through the ulcer, osteomyelitis may be an aggravating complication. An infection can spread into the bone of the foot of a patient with diabetes and/or CN from any infection of adjacent soft tissue that is complicated by an ulcer [9]. Risk factors for osteomyelitis include peripheral neuropathy, vascular disease, limited joint mobility, foot deformities, abnormal foot pressures, minor trauma, a history of ulceration or amputation, and immunosuppression [9–12].

Treatment of early stage CN with the use of crutches and/or immobilization (i.e., total contact cast and/or orthosis) may stop the progression of deformity and reduce the occurrence of complications [13–16]. However, if the diagnosis is initially missed, or if treatment is not initiated, the neuro-osteoarthropathy results in progressive deformities, consecutive ulcers and osteomyelitis, and is accompanied by a high risk of amputation [17].

Several studies have recently demonstrated that the location of diabetic foot ulcers or osteomyelitis affects prognosis and healing time [18–24]. However, there is a paucity of data on healing outcomes of osteomyelitis in the presence of CN. Dalla Paola et al. [25] evaluated the rate of limb salvage and time to recovery in 33 patients affected by CN complicated by diffuse osteomyelitis. However, their study focused on outcomes of surgical treatment to stabilize and correct bone deformity, rather than on outcomes of treating the osteomyelitis. The purpose of this study was to evaluate the treatment of osteomyelitis of the foot in the presence of CN. We hypothesized that there was a difference in (1) amputation rate, (2) level of amputation, (3) duration of antibiotic therapy, and (4) duration of immobilization after treatment in a CN foot with osteomyelitis within versus outside the Charcot zone. The potential effects of the initial surgical treatment, duration of insulin dependency, and patient compliance with treatment on these outcomes were also evaluated.

## Methods

A retrospective analysis of all medical records of patients treated for a diagnosis of CN and osteomyelitis of the same foot between 2002 and 2012 at the outpatient clinic of a large, urban, orthopedic, university-affiliated research hospital was performed. Inclusion criteria were: a diagnosis of CN according to the definition and diagnostic criteria of the French neurologist J.M. Charcot [26], radiographs of the affected foot, and osteomyelitis of the same side with radiological findings of osteomyelitis on MRI and/or positive bone biopsy cultures. Exclusion criteria were: primary treatment at another institution, or a previous fracture due to trauma of the same foot. This study was approved by the Research Ethics Committee of our institution.

Cases were divided into two groups according to whether the osteomyelitis was localized outside the active Charcot region of the foot (Group 1), or within the active Charcot region of the foot (Group 2). The region of the foot (i.e., forefoot, midfoot or hindfoot) where the osteomyelitis was localized was also recorded. To evaluate the effectiveness of the initial surgical treatment, the surgical management was divided into four categories: 1) “limited resection” was defined as resection of the infected bone, leaving the surrounding soft tissue in place, 2) “amputation” was defined as surgical removal of part of the lower limb (bone and soft tissue), 3) “arthrodesis” was defined as removal of the infected bone combined with external (Ilizarov fixateur) or internal fixation, and 4) “debridement” was defined as surgical removal of the infected or necrotic tissue around the wound with underlying osteomyelitis. Successful treatment was defined as the absence of clinical or radiological signs of a recurrence of osteomyelitis at the initially affected region. Duration of treatment was defined as the time from the first clinic visit to the last clinic visit for a single case of osteomyelitis. Antibiotic therapy was discontinued based on the recommendations of in-house infectious disease specialists, the elimination of clinical signs of infection (e.g. redness, warmth and swelling), C-reactive protein level and MRI results. The duration of antibiotic treatment, duration of immobilization, and duration of treatment were calculated in days, separately for each case of osteomyelitis. Other factors likely to influence outcome were recorded, including duration of diabetic treatment, insulin dependency, duration of surgery, smoking status, immunosuppressive therapy, peripheral arterial occlusive disease, obesity, age, gender, incidence of bilateral CN, and patient compliance.

### Statistical analysis

Continuous data are reported as means and standard deviations. Categorical data are reported as numbers and percentages. Statistical analysis was performed using the software R (The R Foundation for Statistical Computing, Version 3.1.0, Vienna, Austria). Differences in categorical baseline characteristics were evaluated using the Mann-Whitney U test and the chi-square test. To address clustering of cases within patients, logistic regression analysis was performed, with amputation as the dependent variable and localization of osteomyelitis as the independent variable with robust standard error (patient identification as a cluster). Duration of antibiotic therapy and duration of immobilization were analyzed as logarithmic transformed dependent variables in linear regression with robust standard error (patient identification as a cluster). Categorical data (i.e., amputation level) were assessed using Fisher’s exact test. The significance level was set at *p* < 0.05. For graphical visualization, Tukey boxplots were depicted with whiskers maximum of 1.5 interquartile ranges (IQR).

## Results

### Study population

This retrospective chart review identified 70 patients diagnosed with CN and osteomyelitis of the same foot between 2002 and 2012. Thirty patients were excluded from the study due to incomplete medical reports (*n* = 26) or absence of reliable evidence of osteomyelitis (*n* = 4). Three patients (#7, #26, and #34) had bilateral osteomyelitis combined with CN. Thus, 40 patients (43 ft) were included in the study (Table 1).

Due to the progressive nature of CN, some patients were successfully treated for multiple episodes of osteomyelitis, which occurred independently of each other, at different sites on the same foot, or at different time points, months or years apart. One patient (#22) presented with osteomyelitis within the Charcot region, and then one month later presented with a separate case of osteomyelitis outside the Charcot region. Ten patients (#1, #4, #7 right foot, #11, #18, #25, #26 left foot, #27, #28, #39) had 2 separately treated and resolved cases of osteomyelitis on the same foot, months apart. Three patients (#6, #7 left foot, #21) had 3 separately treated and resolved cases of osteomyelitis on the same foot, months apart. The mean duration between independent cases of osteomyelitis in a single patient, measured as the date treatment ended for the first case to the date of diagnosis of the second case, was 16.3 months (range: 2.4 to 33.5 months).Thus, in total, we identified 60 cases of osteomyelitis for the 40 patients included in this study. The 60 cases were divided into two groups according to the localization of the osteomyelitis and CN, with 30 cases in Group 1 (i.e., osteomyelitis outside the active Charcot region), and 30 cases in Group 2 (i.e., osteomyelitis within the active Charcot region of the foot).

Patient demographic characteristics and individual treatments are summarized in Table 1. There were 29 (73%) male and 11 female patients; 44/60 (73%) cases of osteomyelitis were in male patients. Mean age was 61.6 ± 12.4 (range, 40.1 to 87.5) years. Male patients (mean age 58.2 ± 10.5; range, 40.1 to 77.5 years) were significantly younger than female patients (mean age 70.4 ± 13.4; range 51.4 to 87.5 years; *p* = 0.02). Twenty-three (58%) patients were between 50 and 70 years of age at initial diagnosis of CN; 8 (20%) were over 70 years of age, and 9 (23%) were under 50 years of age. No female patient was under 50 years of age at initial diagnosis of CN.

Twenty-five of 40 (63%) patients had insulin-dependent diabetes and 15 had non-insulin-dependent diabetes. The mean duration of treatment for diabetes at the time of initial treatment for osteomyelitis was 17.7 ± 13.2 (range: 0.2 to 52.9) years. The mean duration of treatment for diabetes at the time of initial diagnosis of CN was 15.5 ± 13.8 (range: 0.6 to 52.9) years. In Group 1, 16/30 (53%) cases were insulin-dependent; in Group 2, 19/30 (63%) cases were insulin-dependent.

There was no significant difference in age, gender, and duration of treatment for diabetes between the groups (*p* = 0.81, *p* = 0.82, and *p* = 0.30, respectively) (Table 2).

### Amputation rate

An amputation was performed in 31/60 (52%) cases of osteomyelitis (26/40 patients; 26/43 ft). In the 44 male cases, 21 (48%) amputations were performed. In the 16 female cases, 10 (63%) amputations were performed.

The amputation rate was similar for Group 1 with osteomyelitis outside the Charcot region (19 amputations; 63%) and Group 2 with osteomyelitis within the Charcot region (12 amputations; 40%) (*p* = 0.09). Amputation rate did not differ significantly based on insulin dependency or compliance with treatment.

### Level of amputation

**Fig. 1:**
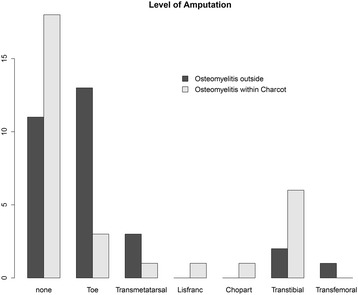
Histogram showing the level of amputation in Group 1 with osteomyelitis outside the Charcot region (*n* = 30) and in Group 2 with osteomyelitis within the Charcot region (*n* = 30) when treatment was successfully completed, and in the absence of recurrence of osteomyelitis

**Fig. 2:**
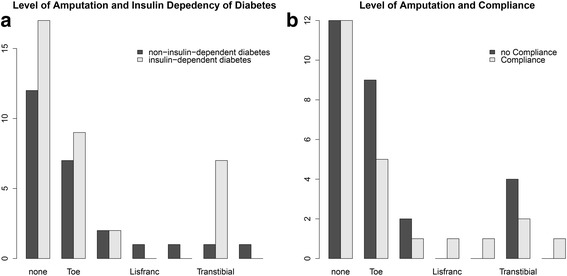
Histograms showing the level of amputation in relation to (**a**) insulin dependency (*n* = 35 insulin-dependent diabetes, *n* = 25 non-insulin-dependent diabetes) and (**b**) compliance with treatment for osteomyelitis and Charcot arthropathy (*n* = 23 compliant, *n* = 27 non-compliant, *n* = 10 not recorded)

The 31 amputations included 16 toe, 4 transmetatarsal, 1 Lisfranc, 1 Chopart, 8 transtibial and 1 transfemoral amputation. A major amputation (i.e., above the level of the ankle) was performed in 3/30 (10%) cases in Group 1 with osteomyelitis outside the active Charcot region and 6/30 (20%) cases in Group 2 with osteomyelitis within the active Charcot region (*p* = 0.009) (Fig. 1). The level of amputation did not differ significantly based on insulin dependency or compliance with treatment (Fig. 2).

### Duration of antibiotic treatment

**Fig. 3:**
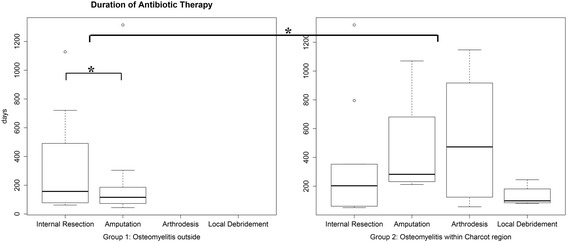
The duration of antibiotic therapy in Group 1 with osteomyelitis outside the Charcot region (mean 55.7 ± 48.9 days) and in Group 2 with osteomyelitis inside the Charcot region (mean 84.1 ± 51.2 days) differed significantly (*p* = 0.045). In Group 1, the duration of antibiotic therapy was 43.9 days shorter in cases of initial amputation compared to cases of initial internal resection (*p* = 0.02). In Group 2, the duration of antibiotic therapy was similar for the different initial surgical treatments (*p* = 0.09). Asterisk = significant difference

The duration of antibiotic treatment was significantly shorter in Group 1 (mean 55.7 ± 48.9, range: 9 to 228 days) compared to Group 2 (mean 84.1 ± 51.2, range: 6 to 238 days, *p* = 0.045). Within Group 1, the mean duration of antibiotic treatment was 43.9 days shorter in cases initially treated with amputation compared to cases initially treated with internal resection (*p* = 0.02). Within Group 2, the duration of antibiotic treatment was similar across all initial surgical treatments (*p* = 0.09) (Fig. 3).

### Duration of immobilization

**Fig. 4:**
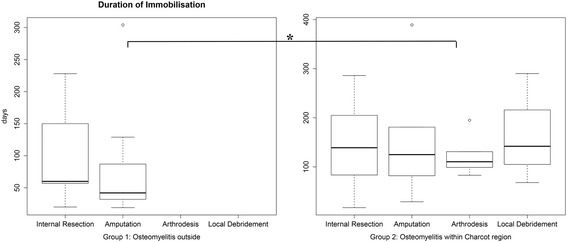
The duration of immobilization in Group 1 with osteomyelitis outside the Charcot region (mean 83.1 ± 70.5, range 19 to 304 days) and in Group 2 with osteomyelitis inside the Charcot region (mean 144 ± 91.8, range 17 to 389 days, *p* = 0.01) differed significantly (*p* = 0.01). The duration of immobilization was similar for the different initial surgical treatments within each group. Asterisk = significant difference

The duration of immobilization was 61.4 days shorter in Group 1 where osteomyelitis outside the active Charcot region (mean 83.1 ± 70.5, range 19 to 304 days), compared to Group 2 with osteomyelitis within the active Charcot region (mean 144 ± 91.8, range 17 to 389 days, *p* = 0.01). There was no significant difference in the duration of immobilization between the different initial surgical treatments within each group (*p* = 0.40 and *p* = 0.90, respectively) (Fig. 4).

## Discussion

Treatment of CN complicated by osteomyelitis is a complex, long-lasting procedure, demanding considerable perseverance from patients and physicians. Multiple surgical procedures, including a high rate of amputations, as well as prolonged antibiotic therapy and immobilization are often required [27]. This study demonstrated that patients treated for osteomyelitis within the Charcot region on the foot underwent more high level amputations and had longer durations of antibiotic treatment and immobilization than patients treated for osteomyelitis outside the Charcot region. However, the amputation rate was statistically similar for both groups.

Amputation was required during the course of treatment in 31/60 cases treated for CN and osteomyelitis of the same foot, whereas 29/60 cases were successfully treated with a combination of conservative surgery and antibiotic medication. The overall amputation rate did not differ significantly between the patients treated for osteomyelitis outside the Charcot region and those treated for osteomyelitis within the Charcot region. Wukich et al. [28] recently reported 16 of 43 patients (37.2%) with CN hospitalized for osteomyelitis underwent major amputation, but this rate can be expected to be lower in less severe cases that can be effectively managed in an outpatient setting such as ours. When considering the level of amputation, the 20% rate of major amputations we reported in cases of osteomyelitis within the Charcot region is comparable to the 23% rate of major amputations reported by Gazis et al. [29] in 47 patients with CN managed by a specialist diabetic foot clinic. However, our 10% amputation rate in cases of osteomyelitis outside the active Charcot region was significantly lower.

The mean duration of antibiotic treatment in this study ranged from 56 to 84 days, where patients with osteomyelitis within the active Charcot region required a longer duration of treatment. Two recent studies reported similar mean durations of antibiotic treatment of 76 days [30] and 77 days [31] for the nonsurgical management of diabetic foot osteomyelitis. Mutluoglu et al. [32] reported a mean of 47 days of antibiotic treatment in 37 patients with diabetic foot osteomyelitis, of whom 22 underwent minor amputation. In 2008, the International Working Group on the Diabetic Foot determined that there are no data to inform the optimal duration of antibiotic therapy [4].

In this study, the mean duration of immobilization ranged from 83 to 144 days, with patients with osteomyelitis within the active Charcot region requiring a longer period of immobilization. Several clinical trials evaluated different off-loading techniques for the treatment of diabetic foot ulcers and reported the duration of immobilization with a total contact cast ranged from 35 to 69 days [33–36]. However, these trials excluded patients with osteomyelitis. In a study of 288 patients with acute Charcot foot that included 81 cases of ulceration and 20 cases of osteomyelitis, the median duration of immobilization for resolution of symptoms was 273 days [37]. Literature reporting specifically on immobilization of the Charcot foot with osteomyelitis is notably lacking.

The region of the foot on which the osteomyelitis was located likely contributed to the significant differences in the amputation level (*p* < 0.001), duration of antibiotic treatment (*p* = 0.045), and duration of immobilization (*p* = 0.01) observed between the groups who presented with osteomyelitis within the Charcot region versus outside the Charcot region. In the group with osteomyelitis within the Charcot region, a major amputation was performed in 3/8 cases with osteomyelitis in the hindfoot, 3/13 cases with osteomyelitis in the midfoot, and none of the cases with osteomyelitis in the forefoot. In the group with osteomyelitis outside the Charcot region, the osteomyelitis was almost exclusively (29/30 cases) located in the forefoot, thus elimination of infection by internal resection or amputation could be achieved more reliably. Diabetic foot ulcers and osteomyelitis located in the forefoot have been demonstrated to have a shorter healing time compared to those in the hindfoot [18, 20–22, 24]. We therefore recommend that patients with osteomyelitis of the foot within versus outside the Charcot region should be analyzed separately in future research evaluating the outcome and treatment of CN and osteomyelitis.

The initial surgical treatment provided in this study did not affect the duration of antibiotic therapy or immobilization in the group with osteomyelitis in the Charcot region, but this may be due to the small sample sizes for each type of surgery. In the group with osteomyelitis outside the Charcot region, the duration of antibiotic therapy was 6 weeks shorter (*p* = 0.02) in those who initially underwent amputation compared to those who initially underwent internal resection, indicating that internal resection may not be sufficient to eliminate the infected tissue.

Four studies recently evaluated limb salvage procedures as alternatives to amputation in Charcot foot and ankle osteomyelitis. Farber et al. [38] reported that none of the 11 patients with midfoot CN and ulceration who underwent operative debridement and corrective osteotomy as a limb salvage procedure needed an amputation. Dalla Paola et al. [39] reported four (9%) major amputations for failed infection control in 43 patients who underwent arthrodesis [40] and external fixation as a limb salvage procedure for CN and ankle osteomyelitis. Pinzur et al. [41] reported 3 amputations (4.2%) in a cohort of 71 patients who underwent single-stage resection of infection and correction of deformity with a ring fixateur. Ramanujam et al. [42] reported one lower extremity amputation (3.7%) in 27 patients with diabetic CN and osteomyelitis who underwent surgical reconstruction using circular external fixation.

A long-standing history (>10 years) of diabetes at the time of initial diagnosis of CN is common [43, 44]. The mean duration of treatment for diabetes in our study was 15 (range: 0.6 to 53) years. No patient with insulin-dependent diabetes received the diagnosis of diabetes less than one year before the diagnosis of CN. However, two patients with non–insulin-dependent diabetes had an almost synchronous diagnosis of CN and diabetes, suggesting that the initial occurrence of Charcot may have guided the diagnosis of diabetes. There were less patients (62%) with insulin-dependent diabetes compared to the 75% previously reported in another study of CN [45]. However, insulin dependency did not appear to influence the amputation rate or amputation level.

In previous studies, noncompliance with treatment of CN was determined to be the strongest predictor for recurrence of CN, with an odds ratio of 19.7 [46, 47]. While the rate of noncompliance as recorded in patient charts was high in the present study (38%), noncompliance with treatment of the osteomyelitis did not appear to influence the amputation rate or level of amputation.

The large number of patients excluded from this study due to incomplete patient charts may have resulted in selection bias. Typically, CN patients do not come in for follow-up visits because they are poorly compliant with treatment (i.e., total contact cast) or they do not understand that they have a disease. The less complex cases are often followed for longer durations than the severe cases, to monitor their feet and their special shoes. Thus, to minimize the potential selection bias for more severe cases, we evaluated the effect of compliance with treatment on amputation rate and amputation level.

Limitations of this study include the retrospective study design, such that some pertinent factors that could potentially affect treatment outcomes may not have been recorded, and data collection at a single site, which limits the generalizability of the conclusions. Another limitation is the lack of recorded reasons for the initial surgical treatment that was elected. Finally, the heterogeneity of initial surgical treatments performed in Group 2 patients and the relatively small sample sizes did not allow further subgroup analysis.

Strengths of this study include the relatively large sample size compared to other studies of CN, and the detailed information available about the treatment regimens. However, the heterogeneity of patients with this disease and initial surgical treatment options limited may require prospective trials with larger samples to better elucidate the best treatment option for osteomyelitis in the presence of CN.

## Conclusions

Patients treated for osteomyelitis within the Charcot region on the foot underwent more high level amputations and had longer durations of antibiotic treatment and immobilization than patients treated for osteomyelitis outside the Charcot region, although the amputation rate was similar for both groups. We recommend that. osteomyelitis outside and within the Charcot affected region should be regarded as separate entities when considering treatment protocols and in future research evaluating the outcome and treatment of CN and osteomyelitis. If osteomyelitis occurs outside the active Charcot region, primary amputation may be preferred to internal resection.

## Abbreviations

CN: Charcot neuroarthropathy
IQR: interquartile range
MRI: magnetic resonance imaging
